# Relative bradycardia in patients with COVID-19

**DOI:** 10.1186/s42444-022-00073-z

**Published:** 2022-09-01

**Authors:** Lae-Young Jung, Jae-Min Kim, Sukhyun Ryu, Chang-Seop Lee

**Affiliations:** 1 Department of Internal Medicine, Jeonbuk National University Medical School, 567 Baekje-daero, Deokjin-gu, Jeonju-si, Jeollabuk-do 54907 Republic of Korea; 2 Biomedical Research Institute of Jeonbuk National University Hospital, Jeonju, Republic of Korea; 3 Department of Preventive Medicine, Konyang University College of Medicine, Daejeon, Republic of Korea

## Abstract

**Introduction:**

Relative bradycardia(RB) is a relatively low heart rate response to rise in body temperature that occurs in several infectious diseases and can be an important clinical sign. In previous case reports, RB was presented in some patients with Coronavirus disease 2019 (COVID-19) COVID-19.

**Objective and Methods:**

To investigate the correlation between temperature and heart rate, we retrospectively reviewed 249 febrile patients with documented COVID-19 patients. RB was defined as a rise in the heart rate from a basal heart rate of less than 10 beats/minute/°C rise in temperature.

**Results:**

In this study, the prevalence of RB in patients with COVID-19 was 60.6%. When the HR at peak temperatures for patients with COVID-19 were compared with reference valve (general temperature-heart rate response in infectious disease), our findings demonstrate a relatively lower heart rate at all peak temperatures recorded. Despite differences in heart rate response, there were not significant differences in clinical outcomes (pulmonary manifestation, intensive care unit admission, Death).

**Conclusions:**

Most patients with COVID-19 are associated with relative bradycardia, not related to clinical outcomes. RB in COVID-19 can be considered as the clinical features for differential diagnosis from other febrile conditions.

## Introduction

Coronavirus disease 2019 (COVID-19) is known to cause variable extra-respiratory manifestations, including the cardiovascular system. Arrhythmia is one of the reported cardiac complications includes variable situations, from nonspecific electrocardiographic changes to serious arrhythmias in critically ill patients [1]. Early studies reported the incidence of arrhythmias in patients with COVID-19 to be as high as 16.7% [2].

Relative bradycardia (RB) is a relatively low heart rate response to rise in body temperature. RB is a clinical term that is often used in daily practice and the literature as a clinical sign for an individual patient and a characteristic of some infectious diseases. Recently, there has also been studies of bradycardia and relative bradycardia in patients with COVID-19 infection [3–5]. Interestingly, bradyarrhythmias including RB were much more common in patients from Asian (up to 40%) compared with other races [6]. However, the enrolled patients were few and the definition of RB was different for each study, raising a question about the real incidence. In addition, no studies have yet been conducted on whether RB can be considered as a clinical feature or its role as a prognostic factor. We, therefore, aimed to evaluate the incidence of relative bradycardia in patients with COVID-19 and to determine potential correlations with the progression.

## Method

We conducted a retrospective medical records’ review of 249 patients (57.59 ± 20.73 years, 49.8% male subjects) serologically diagnosed with COVID-19 by using reverse transcription PCR from February 2020 to June 2021 in a tertiary care referral hospital. Exclusion criteria were as follows: age below 18 years, history of specific arrhythmias (atrial fibrillation, sick sinus syndrome, atrioventricular block), treatment with heart rate-lowering medications (e.g., non-dihydropyridine calcium channel blockers or beta blockers), and bradycardia associated with other specific medical conditions (e.g., electrolyte imbalance). The data were reviewed by trained physicians. The study protocol was reviewed and approved by the institutional review board (IRB) of Jeon-buk National University Hospital (IRB Number; CUH 2022-03-003).

Symptoms, vital signs, laboratory findings, chest images, and treatment during the hospital days were collected. Body temperature was measured with an ear thermometer (Thermoscan IRT 4520; Braun, Kronberg, Germany), and fever was defined as temperature greater than 37.8 °C. Body temperature and heart rate were assessed every 4 h. Maximal temperature was defined as the highest recorded temperature during the hospital stay. Basal temperature was defined as body temperature after the ‘febrile’ period. Basal heart rate was defined as the average heart rate during the last 24 h before discharge. Although there is no uniform definition of RB, we defined it a priori as a rise in the heart rate from a basal heart rate of less than 10 beats/min/°C rise in temperature, as was commonly used in previous studies [7–9]. A pulse increase greater than 10 beats/min/°C was classified as a general heart rate response (GHRR).

Categorical variables were described as frequency and percentages. Fischer’s exact test was used to evaluate categorical variables. All continuous variables are described as means ± SD that were compared using Student’s t test. All analyses were two tailed, with clinical significance defined as *p* < 0.05. All statistical processing was performed using SPSS-PC 25.0 (Statistical Package for the Social Sciences, SPSS-PC. Inc., Chicago, IL).

## Result

**Fig. 1:**
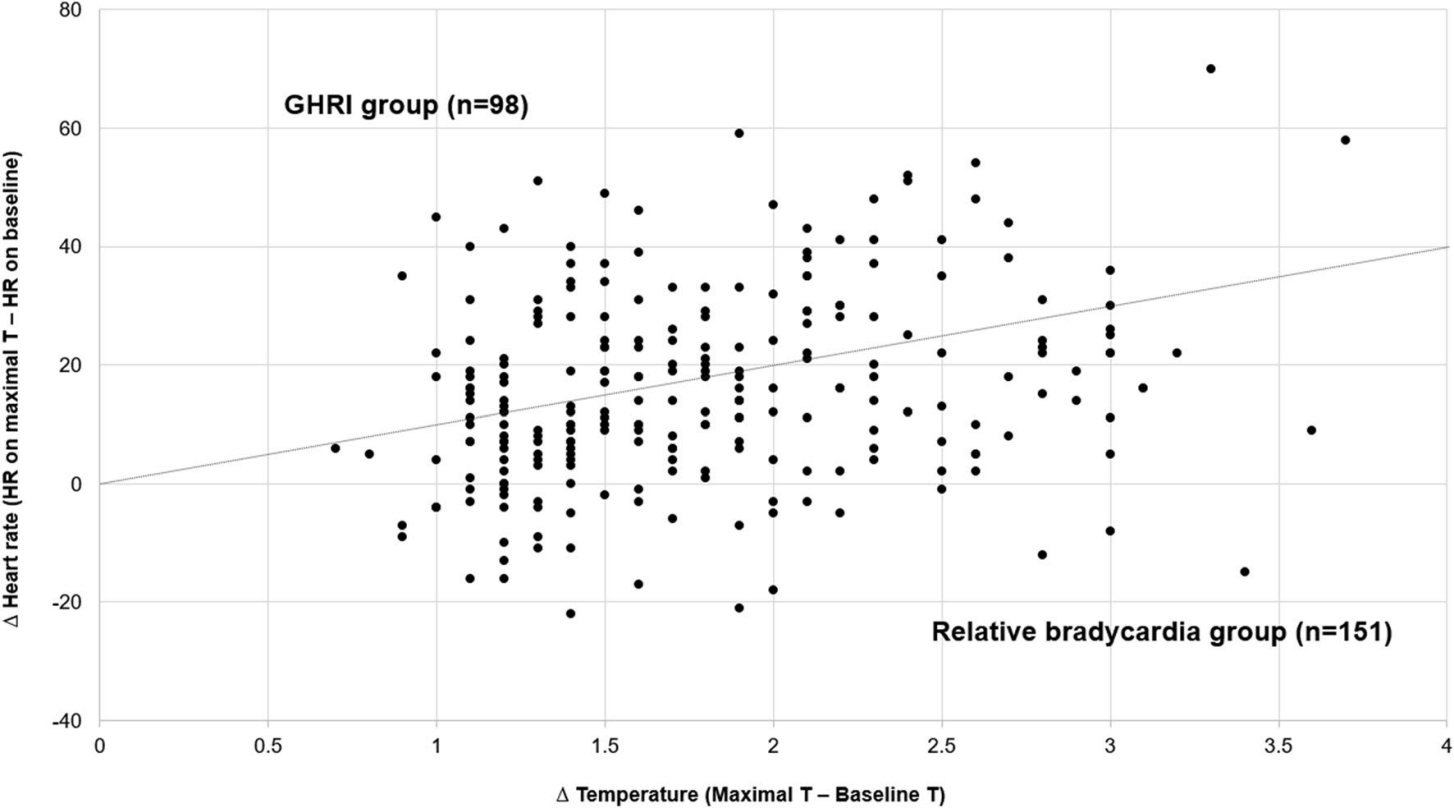
Distribution of Δheart rate/Δtemperature by group

**Fig. 2:**
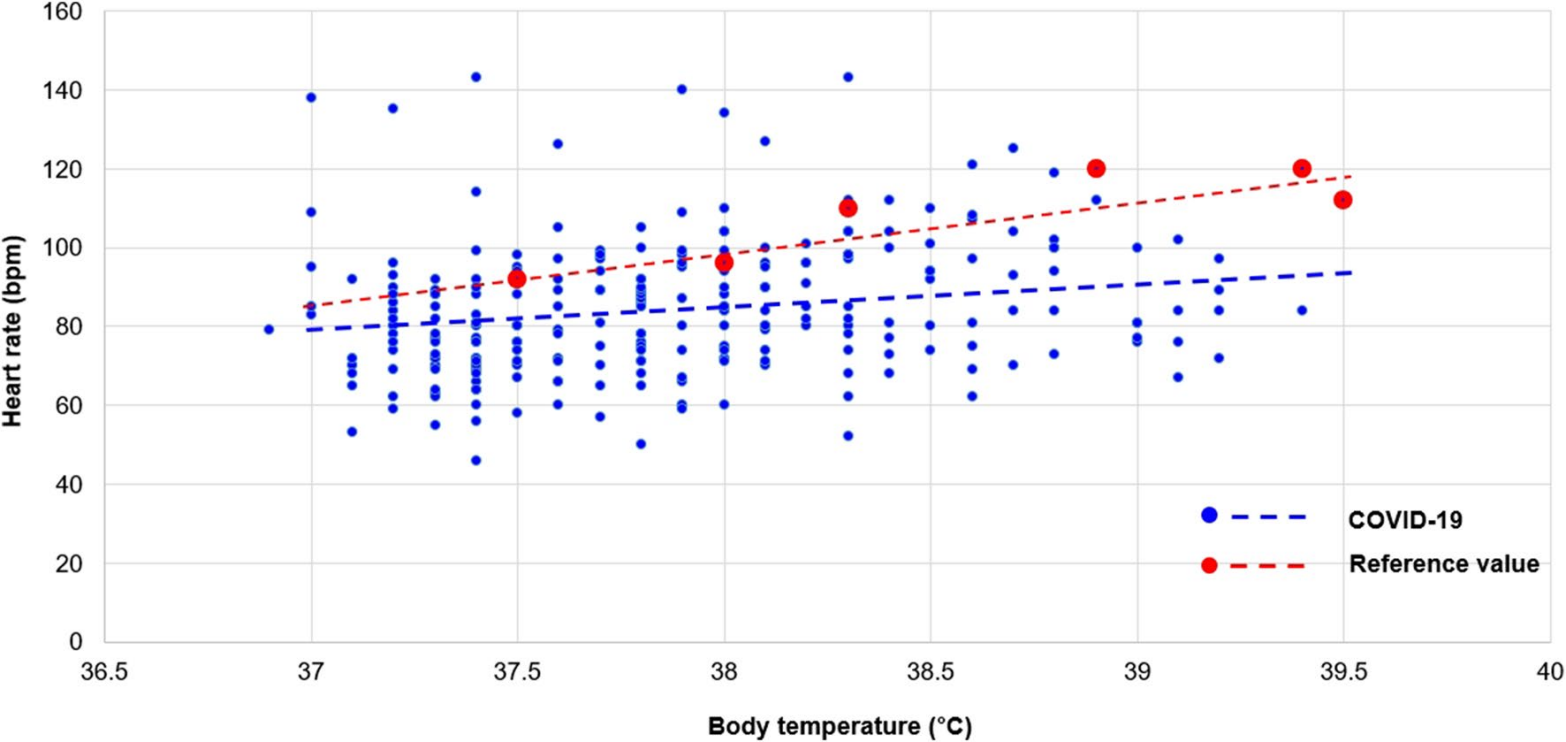
Temperature–heart rate relationship in patients with COVID-19 at peak temperatures (red spot and line: reference value (General relationship in infectious disease) adopted from *Emerging infectious diseases 2007;13:650*. and *infect Dis practice 1997;21:38–40*

**Fig. 3:**
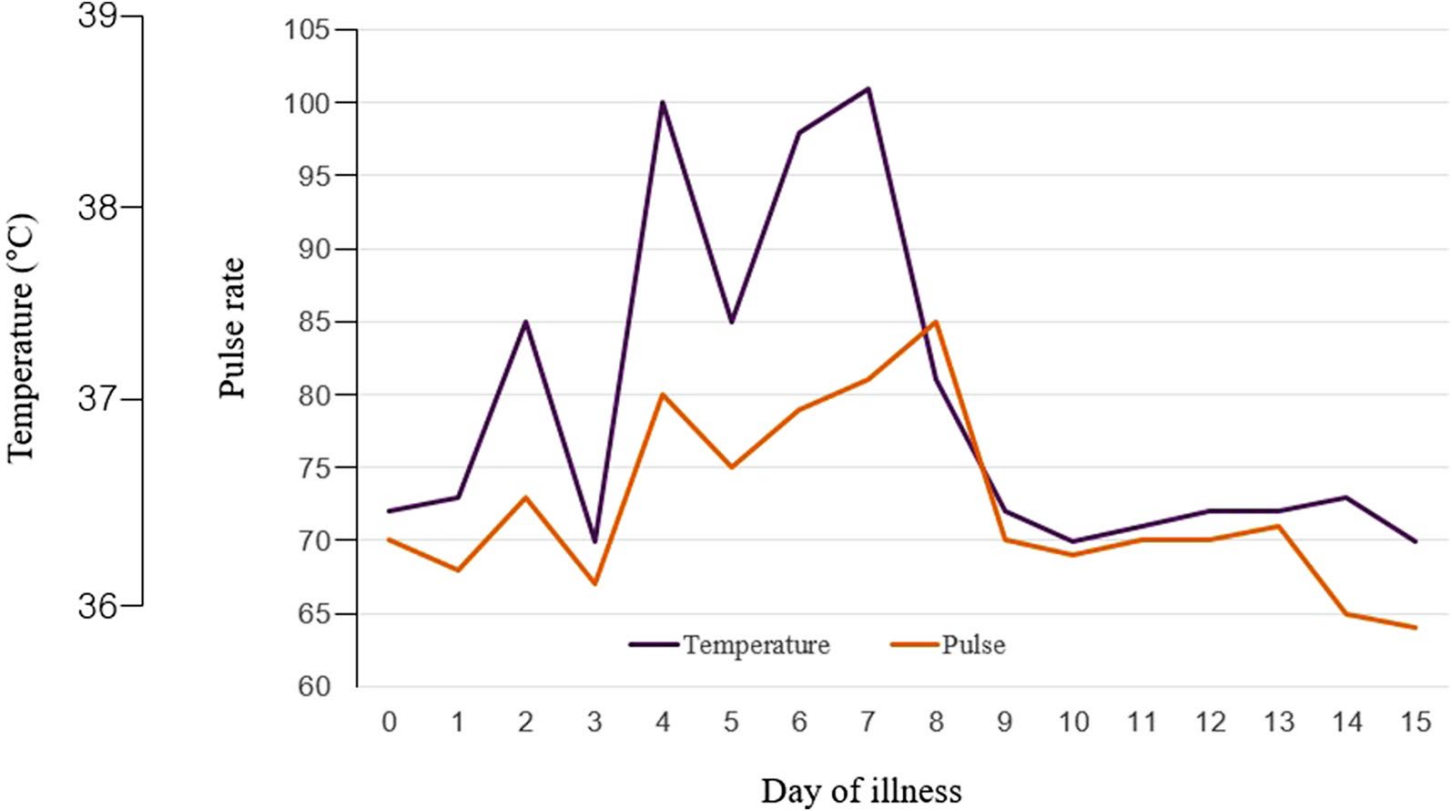
Vital sign chart in patient with COVID-19 (#125, median value)

This study showed the prevalence of RB in patients with COVID-19 was 60.6% (Fig. 1). When the heart rates at peak temperatures for patients with COVID-19 were compared with reference valve (general temperature-heart rate response in infectious disease), our findings demonstrate a relatively lower heart rate at all peak temperatures recorded (Figs. 2 and 3). Patients in the RB group were significantly older (60.24 ± 18.41 years) and were more likely to have diabetes. Baseline laboratory findings and basal temperatures were not significantly different between the two groups (Tables 1, 2). Although, the RB group had a significantly higher median resting heart rate and had a significantly lower heart rate than the GHRR group at maximal temperature (73.53 ± 13.73 vs. 61.83 ± 11.06; *p* < 0.001), the opposite phenomenon was seen during maximal temperature, with relative bradycardia group achieving significantly lower heart rates than the GHRR group (78.91 ± 14.36 vs. 93.63 ± 17.12; *p* < 0.001) (Table 2). Despite differences in heart rate response, no significant differences were seen in clinical outcomes (pulmonary manifestation, intensive care unit (ICU) admission, death) (Table 3).

**Table 1:** Baseline clinical characteristics and laboratory findings

	RB (*n* = 151)	GHRR (*n* = 98)	*p* value
*Clinical characteristics*			
Gender (male)	77 (51.0%)	47 (48.0%)	0.698
Age	60.24 ± 18.41	53.52 ± 23.47	0.018
Diabetes	40 (26.5%)	15 (15.3%)	0.038
Hypertension	64 (42.4%)	33 (33/7%)	0.169
CKD	9 (6.0%)	9 (9.2%)	0.609
COPD	10 (6.7%)	4 (4.1%)	0.389
Malignancy (recent)	7 (4.7%)	1 (1.0%)	0.115
*Laboratory findings at admission*			
WBC (× 10^3^/µL)	5.51 ± 2.83	6.16 ± 3.94	0.172
Hb (g/dL)	13.21 ± 1.67	13.18 ± 1.82	0.911
Hct (%)	38.91 ± 4.70	39.31 ± 6.66	0.583
Platelet (× 10^3^/µL)	153.50 ± 68.78	211.84 ± 74.15	0.082
PT (s)	12.33 ± 2.23	12.34 ± 1.67	0.985
AST (IU/L)	44.40 ± 27.57	42.87 ± 28.39	0.701
ALT (IU/L)	34.44 ± 27.66	36.43 ± 31.34	0.605
Total bilirubin (mg/dL)	0.63 ± 0.37	0.63 ± 0.31	0.966
Albumin (g/dL)	4.25 ± 0.47	4.18 ± 0.55	0.279
Creatinine (mg/dL)	0.97 ± 1.29	0.86 ± 0.46	0.444
CRP (mg/L)	49.35 ± 59.34	55.41 ± 66.46	0.466

**Table 2:** Alternation of body temperature and heart rate

	RB (*n* = 151)	GHRR (*n* = 98)	*p* value
Basal temperature (°C)	36.07 ± 0.22	36.10 ± 0.23	0.380
Maximal temperature (°C)	37.90 ± 0.59	37.85 ± 0.49	0.480
Basal heart rate	73.53 ± 13.73	61.83 ± 11.06	< 0.001
Heart rate at maximal temperature	78.91 ± 14.36	93.63 ± 17.12	< 0.001

**Table 3:** Clinical outcomes

	RB (*n* = 151)	GHRR (*n* = 98)	*p* value
Pulmonary involvement on radiologic test	103 (68.2%)	68 (69.4%)	0.889
Steroid therapy	50 (33.1%)	40 (40.8%)	0.216
ICU admission	35 (23.3%)	29 (29.6%)	0.300
Death during hospital days	8 (5.3%)	3 (3.1%)	0.534

## Discussion

In this study, prevalence of RB in patients with COVID-19 was 60.6%. This rate was higher than previous reports. Capoferri et al. reported 110 hospitalized COVID-19 patients in which 36% developed relative bradycardia and of the patients with a fever 56% developed relative bradycardia [4]. Our definition of a relative bradycardia, a rise in heart rate from a basal heart rate of less than 10 beats/min/°C rise in temperature represents the lower border of the general febrile heart rate response of 10–18 beats/min/°C during infectious conditions [10, 11]. However, the application of alternative definitions of relative bradycardia would greatly affect our results. For example, a previous study defined relative bradycardia as an increase in pulse rate < 18 beats/min for each 1 °C increase in body temperature [5]. The prevalence of relative bradycardia in our population increased from 60.6 to 76.3% when this cut-off value was used. The definition of relative bradycardia was different in the previous two studies [4, 5]. The definition used in this study is the same definition of RB used in the diseases in which RB is accepted as a specific clinical feature (typhoid fever, scrubs typhus) [12]. Going forward, and for future studies, we suggest an unambiguous and unified definition be agreed upon.

The impact of COVID-19 infection on the cardiovascular system and its connection with bradycardia is likely multifactorial, and varies with disease severity as well as clinical setting. One of the most popular theories stems to the association of coronavirus and the angiotensin-converting enzyme 2 receptors [13]. It is likely that coronavirus has an inherent ability to invade the myocardial tissue. Although the mechanism of relative bradycardia is unclear, a hypothesis is that direct pathogen effects on the sinoatrial node and increased levels of inflammatory cytokines, such as interleukin-6, which was reported for patients with COVID-19, can increase vagal tone and decrease heart rate variability [14].

RB is a clinical term that is often used in daily practice and the literature as a clinical sign for an individual patient and a characteristic of some infectious diseases. The term has been defined in several studies [7] and can be an important diagnostic finding for variety of infectious diseases including Legionnaires’ disease, typhoid fever, psittacosis, typhus, leptospirosis, malaria, and babesiosis. RB may be used to differentiate among infectious diseases in specific clinical situations. Because the prevalence of RB in patients with COVID-19 was up to 60.6% in this study, it could also be used as a specific clinical feature to differentiate among similar infectious conditions.

In this study, basal heart rate was higher in the RB group than the GHRR group whereas the maximal heart rate was higher in the GHRR group. The RB group was older than GHRR group. These findings were consistent with previous studies, which presented that most cases occurred in patients over the age of 65 [15]. The RB did not seem to affect clinical outcomes in this study. This result may suggest that RB caused by COVID-19 does not mean critical cardiac manifestation.

Several limitations were present in this study. First, half of patients received antipyretic medicines during their hospitalization (acetaminophen was most commonly used). Because fever was possibly underestimated for these patients, relative bradycardia might be a more common clinical sign. Second, it was a study of patients admitted to tertiary hospitals, and many of them presented with severe conditions. Hence, the results may be different in more general patients. Moreover, relative bradycardia is a poorly defined clinical term, and further studies are needed to investigate its role in the diagnosis of COVID-19.

In conclusion, most patients with COVID-19 are associated with RB, not related to clinical outcome. RB in COVID-19 can be considered as the clinical features for differential diagnosis from other febrile conditions.

## Data Availability

The datasets used and analyzed during the current study are available from the corresponding author on reasonable request.

## Abbreviations

RB: Relative bradycardia
COVID-19: Coronavirus disease 2019
ICU: Intensive care unit
IRB: Institutional review board
GHRR: General heart rate response
