# Correction: Relative bradycardia in patients with COVID-19

**DOI:** 10.1186/s42444-022-00083-x

**Published:** 2023-01-09

**Authors:** Lae-Young Jung, Jae-Min Kim, Sukhyun Ryu, Chang-Seop Lee

**Affiliations:** 1 Department of Internal Medicine, Jeonbuk National University Medical School, 567 Baekje-daero, Deokjin-gu, Jeonju-si, Jeollabuk-do 54907 Republic of Korea; 2 Biomedical Research Institute of Jeonbuk National University Hospital, Jeonju, Republic of Korea; 3 Department of Preventive Medicine, Konyang University College of Medicine, Daejeon, Republic of Korea

**Correction: International Journal of Arrhythmia (2022) 23:22** 10.1186/s42444-022-00073-z

The abstract was missing from the original publication; the abstract is provided in this correction article. The original article has been updated.

**Abstract**

**Introduction** Relative bradycardia (RB) is a relatively low heart rate response to rise in body temperature that occurs in several infectious diseases and can be an important clinical sign. In previous case reports, RB was presented in some patients with Coronavirus disease 2019 (COVID-19).

**Objective and methods** To investigate the correlation between temperature and heart rate, we retrospectively reviewed 249 febrile patients with documented COVID-19 patients. RB was defined as a rise in the heart rate from a basal heart rate of less than 10 beats/minute/°C rise in temperature.

**Results** In this study, the prevalence of RB in patients with COVID-19 was 60.6%. When the HR at peak temperatures for patients with COVID-19 was compared with reference valve (general temperature–heart rate response in infectious disease), our findings demonstrate a relatively lower heart rate at all peak temperatures recorded. Despite differences in heart rate response, there were not significant differences in clinical outcomes (pulmonary manifestation, intensive care unit admission, death).

**Conclusion** Most patients with COVID-19 are associated with relative bradycardia, not related to clinical outcomes. RB in COVID-19 can be considered as the clinical features for differential diagnosis from other febrile conditions.

